# Use of a 3D-Printed Patient-Specific Surgical Jig and Ready-Made Total Sacral Endoprosthesis for Total Sacrectomy and Reconstruction

**DOI:** 10.1155/2021/3250002

**Published:** 2021-12-13

**Authors:** Qianyu Shi, Tao Ji, Siyi Huang, Xiaodong Tang, Rongli Yang, Wei Guo

**Affiliations:** Musculoskeletal Tumor Center, People's Hospital, Peking University, Beijing, China

## Abstract

**Objective:**

In the present study, the authors aimed to optimize the workflow of utilizing a 3D printing technique during surgical treatment for malignant sacral tumors, mainly on preparation of patient-specific surgical jigs and ready-made 3D-printed total sacral endoprosthesis.

**Methods:**

Three patients with a malignant sacral tumor received total sacrectomy with preoperative design of a patient-specific 3D-printed cutting jig and endoprosthetic reconstruction. Size of ready-made 3D-printed endoprosthesis was determined based on preoperative images, planned surgical margin, and size of the endoprosthesis. A patient-specific cutting jig was designed with a bilateral cutting slot matching the bilateral planes of the implant precisely. The tumor was removed en bloc through a single posterior approach only, being followed by reconstruction with ready-made total sacral endoprosthesis.

**Results:**

The mean time for preoperative design and manufacture of the surgical jig was 6.3 days. Surgical jigs were successfully used during surgery and facilitated the osteotomy. The mean operation time was 177 minutes (range 150-190 minutes). The mean blood loss was 3733 ml (range 3600-4000 ml). R0 resections were achieved in all the three cases proven by pathology. Evaluation of osteotomy accuracy was conducted by comparing preoperative plans and postoperative CT scans. The mean osteotomy deviation was 2.1 mm (range 0-4 mm), and mean angle deviation of osteotomy was 3.2° (range 0-10°). At a mean follow-up of 18.7 months, no local recurrence was observed. One patient had lung metastasis 15 months after surgery. Two patients were alive with no evidence of the disease.

**Conclusions:**

The patient-specific surgical jig and ready-made 3D-printed total sacral endoprosthesis can shorten the surgical preparation time preoperatively, facilitating accurate osteotomy and efficient reconstruction intraoperatively. The workflow seems to be feasible and practical.

## 1. Introduction

Primary malignant sacral neoplasms are comparatively rare [1]. Among these, osteosarcoma, Ewing's sarcoma, and chordoma are relatively common. The tumors are prone to relapse after conventional therapy such as chemotherapy and radiation therapy [2, 3], thus making the surgical excision with negative margins of great importance. Nevertheless, the complexity of local anatomy and the difficulty of attaining R0 resection make the local recurrence rate not satisfying [4]. The en bloc sacrectomy accompanied by sufficient tumor-free margin is needed for diminishing the local recurrence and improve the prognosis of patients.

Tumors in the sacral area are difficult to remove due to the intricate anatomy, which aggravates the difficulty of achieving the R0 resection. Also, the high risk of intraoperative massive hemorrhage adds technical challenges during surgical removal [5–7]. Intralesional surgery of the primary malignant sacral tumor is not recommended because of its high recurrence rate and poor prognosis. Kaiser et al. reported that the local recurrence rate after R0 resection (28%) was significantly lower than that of intralesional resection (64%) [8]. Total sacral resection is used to achieve better local control for primary malignant tumors involving the higher sacrum. One of the major challenges during en bloc sacrectomy was the reconstruction of spinopelvic continuity after resection. The reconstruction methods were varied and not well established yet vital to the fixation of the lumbosacral region, posterior pelvic ring, and anterior spinal column [6, 9–11].

**Figure 1:**
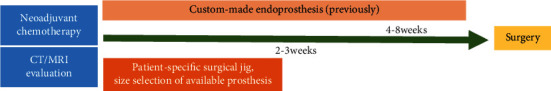
Optimized workflow of utilizing the 3D printing technique during surgical treatment for malignant sacral tumors. The flowchart showed the decreased surgical preparation period preoperatively from 4-8 weeks previously to 2-3 weeks currently.

The reconstruction method using the screw-rod system was not satisfying for its late complications, especially high mechanical failure rate. 3D printing prosthesis could overcome those shortcomings. The advantages of using a 3D printing total sacral endoprostheses included the following: (1) the prosthesis reconstructed the lumbosacral and pelvic ring in one step by integrating spinal pelvic fixation (SPF), posterior pelvic ring fixation (PPRF), and anterior spinal column fixation (ASCF) and (2) the metallic porous surface facilitated bone ingrowth thus enhancing stability in the long term, but the period of manufacturing time was usually consuming, usually 4-8 weeks, which was impractical for patients with malignant tumors, especially for those who received neoadjuvant chemotherapy. The optimal interval between neoadjuvant chemotherapy (osteosarcoma and Ewing's sarcoma) and surgery is about 2~3 weeks. In order to shorten the waiting time preoperatively, a new workflow (Figure 1) was established to optimize the preoperative preparation including design and manufacture of a patient-specific osteotomy jig and size selection of a ready-made 3D-printed total sacral endoprosthesis.

**Figure 2:**
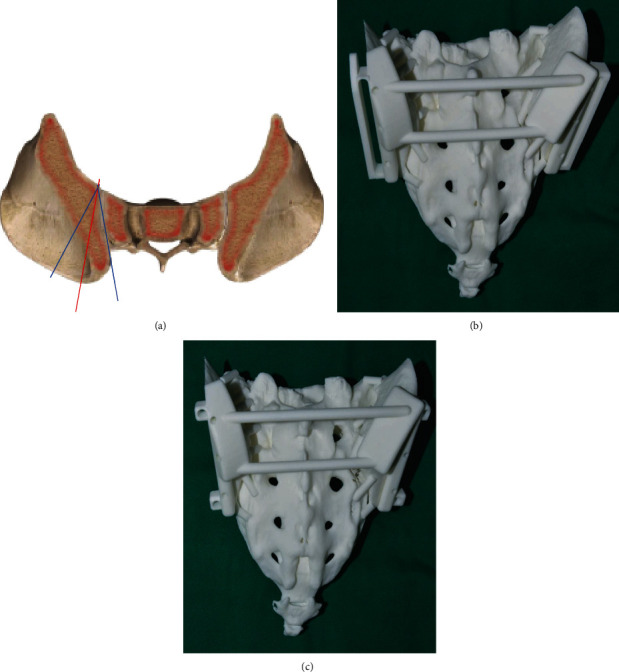
Inaccurate osteotomy by Gigli saw and patient-specific surgical jig model. The red line indicated the planned osteotomy line, while blue was normally carried out during surgery. The models showed two types of patient-specific surgical jig: (b) the oscillating saw type and (c) Gigli saw type.

For total en bloc sacrectomy, the bilateral osteotomy sites within the ilium determined not only the surgical margin but also the accurate of bone-implant contact. The site and direction of osteotomy determined whether the bone cutting surface can perfectly contact with the endoprosthesis [12]. Usually, a Gigli saw was used to perform osteotomy during total sacrectomy by a single posterior approach. Precise placement of a Gigli saw and performing osteotomy with accurate direction were extremely difficult around the sacroiliac joint (Figure 2(a)). The patient-specific cutting jig is an emerging application of a three-dimensional (3D) printing technique, which is becoming a well-accepted method for osteotomy due to its better visualization, precise orientation, and less long-term complications [13–16]. Preoperative CT and MRI determined the osteotomy sites and size selection of ready-made 3D-printed prosthesis. The bilateral bone-contact surface of the 3D-printed prosthesis was the reference for the site and direction of osteotomy, which was essential during the design of the patient-specific surgical jig. Therefore, optimizing the workflow including design and manufacture of a patient-specific surgical jig and size selection of a ready-made 3D-printed prosthesis may improve accuracy of surgical resection and endoprosthetic reconstruction with precise bone-implant surface contact intraoperatively with an acceptable and practicable preoperative surgical preparation interval.

In this present study, we describe the workflow of preparation of a patient-specific surgical jig and selection of a ready-made 3D-printed prosthesis preoperatively for en bloc sacrectomy and reconstruction. Three patients of sacral tumor underwent total en bloc sacrectomy with the use of a patient-specific surgical jig, followed by 3D-printed prosthesis reconstruction, aimed at optimizing the workflow of utilizing 3D printing technology in resection and reconstruction after total en bloc sacrectomy.

## 2. Methods

### 2.1. Preoperative Workflow

**Figure 3:**
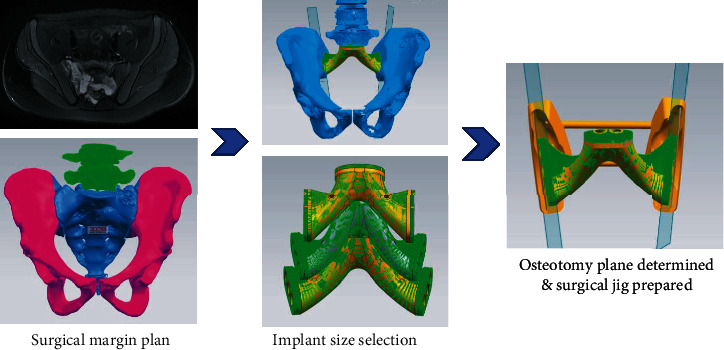
Current preoperative planning. The surgical margin was designed based on the images after neoadjuvant chemotherapy. Then, size of ready-made total sacral endoprosthesis was selected accordingly. The surgical jig was designed with osteotomy planes being matched to bilateral surfaces of prosthesis precisely. Anatomical contour of the posterior iliac spine was used as the placement reference of the jig intraoperatively.

3D-printed total sacral endoprostheses were started being used at the authors' institution since 2015 [15], and the main advancement of the endoprosthetic design compared with the screw-rod system was the monobloc design recovering three main structures at the lumbosacral region along with porous bone-implant interfaces. There were three commercial available sizes to choose intraoperatively, so no additional waiting time before surgery was needed for endoprosthesis manufacture. However, without a surgical jig, we were facing difficulties of the unpredicted match of the bone-implant surface at bilateral iliac osteotomy sites during surgery. For cases that a custom-made endoprosthesis was needed due to defect beyond three sizes, a longer preoperative preparation time was needed, usually 4-8 weeks. In order to optimize the precision of osteotomy and improve match of bone-implant surfaces, we prepared a patient-specific surgical jig preoperatively. Also, size selection of endoprosthesis was conducted based on the extent of the tumor with adequate surgical margins. Preoperative CT and MRI were required after neoadjuvant chemotherapy for osteosarcoma and Ewing's sarcoma, and the osteotomy site was determined on both the axial sections and virtual 3D model. Then, a certain size of 3D-printed total sacral endoprosthesis was chosen to reconstruct the defect on the digital 3D model. A patient-specific surgical jig (AK Medical) was designed and prepared to facilitate osteotomy (Figure 3). The osteotomy planes were precisely matched to the bilateral surfaces of the endoprosthesis. There were three main design elements of the surgical jig (Figures 2(b)and 2(c)): (1) matching region: contour feature of the bilateral posterior iliac spine (PIS) and distance between two PIS were used to develop the surface matching area in the jig; (2) fixation holes: there were three 3.0 mm holes on each side for Kirschner wires to stabilize the surgical jig during osteotomy; and (3) osteotomy slots: the planes of osteotomy was designed according to the bilateral surface of the selected endoprosthesis, and also the depth of the cutting slot was measured preoperatively. The two types of design can facilitate either the Gigli saw (two-whole passing Gigli saw) or oscillating saws (groove design). Institutional review board approval and patient consent were obtained prior to the initiation of the study.

The 3D-printed prosthesis was designed based on an anatomical database of patients who had undergone total sacrectomy as reported previously [12, 13, 15, 17]. The design derived from the concept of a metal prosthesis with porous bone-implant interfaces that could connect the lumbar spine and ilium, connect both sides of the ilium, and reconstruct the structure of loading transfer through the anterior spinal column in one step and be conducive to bone ingrowth as well. Consisting of three contacting surfaces, the prosthesis could reconstruct the stability of the lumbarsacral joint and bilateral sacroiliac joints, which was accomplished by the contact of the proximal surface and the inferior endplate of L5 vertebrae and the contact of surfaces on both flanks and bilateral iliac osteotomic planes, respectively. Three sizes of prosthesis were available to fit the defect during surgery. A patient-specific surgical jig was designed based on the anatomic characteristics of the posterior iliac crest and the bilateral faces of prosthesis. The expected osteotomy planes and matching accuracy of prosthesis were tested on a sacral model to verify the accuracy preoperatively (Figures 2 and 3).

### 2.2. Surgical Procedures

**Figure 4:**
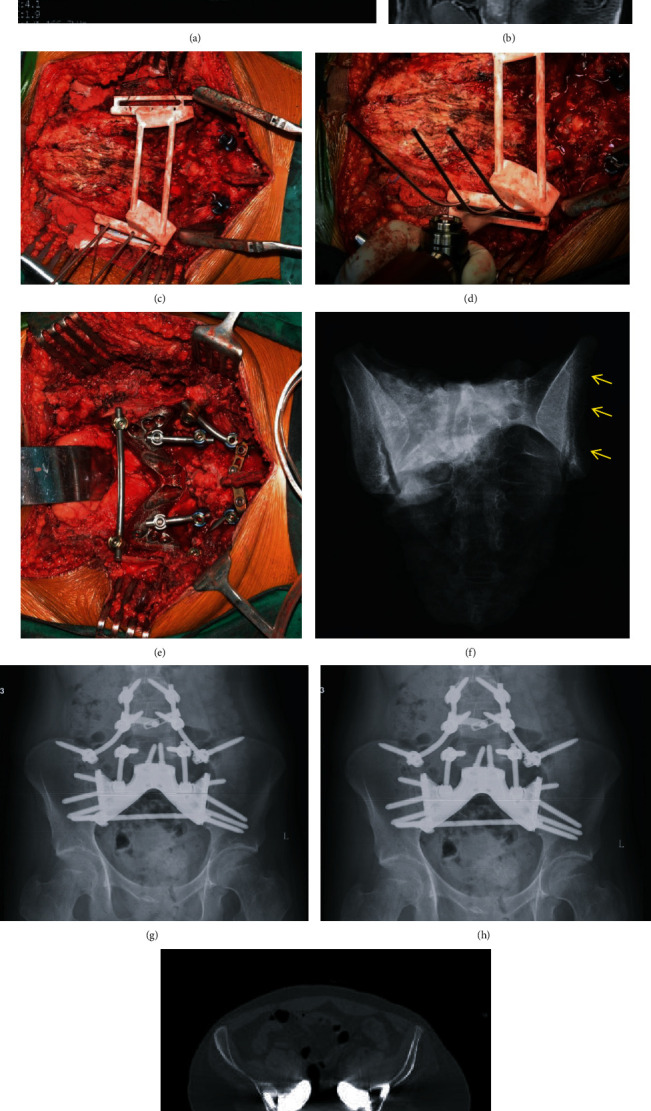
An illustrative case of sacral osteosarcoma. Patient no. 1. The MRI showed the extent of tumor involvement (a, b). The surgical jig was placed after exposure of the posterior sacrum along with the bilateral posterior iliac spine, being stabilized by K-wires (c). The direction of wires was parallel to the planned osteotomy planes. Then, the oscillating saw was used to complete the osteotomy (d). Intraoperative photo showed reconstruction with a screw-rod system and 3D-printed total sacral endoprosthesis (e). Specimen X-ray (f): arrows indicated the K-wire fixation holes. Follow-up at one year (g) and 2 years (h) revealed no complication of the implants. The axial CT scan verified the perfect contact of bone-implant (i).

Usually, preoperative selective artery embolization was performed to decrease intraoperative hemorrhage, and an aorta balloon catheter was used to temporally block the arterial blood flow intraoperatively [6]. Prone position was used, and an inverted Y-shape incision was utilized. Dissection and exposure were performed as reported previously [15]. After the posterior of the sacrum and bilateral PIS were exposed, the surgical jig was placed on the PIS with contour matching. Once placement of the jig was in position, it was stabilized by Kirschner wires. The oscillating saw was used for osteotomy at the planned site with specific direction (intraoperative video as supplemental file (available here)). The sacrum and bilateral partial ilia were removed with adequate margin (Figure 4). The selected size of ready-made 3D-printed prosthesis was used to reconstruct the bone defect (Figure 4(e)). Fixation screws were placed into the vertebrae of L5 and bilateral ilium. Two rods were used to connect the lower lumbar pedicle screws with the prosthesis. The mean operation time was 177 minutes (range 150-190 minutes), and the mean estimated intraoperative blood loss was 3733 ml (range 3600-4000 ml).

### 2.3. Postoperative Care

Drainages were removed once the daily volume was less than 50 ml and prophylactic antibiotics were stopped. The patients were restricted to bed rest for 4-6 weeks with Flowler's position allowed. Weight-bearing was authorized after bed rest.

## 3. Results

The diagnosis of the three cases was osteosarcoma, Ewing's sarcoma, and chordoma each. The mean age was 26 years old. The mean time for preoperative design and manufacture of the surgical jig was 6.3 days. Surgical jigs were successfully used during surgery and facilitated the osteotomy. R0 resections were achieved in all the three cases. Through the comparison between postoperative CT scans and preoperative expected osteotomy planes, the errors of the procedure are mean osteotomy deviation 2.1 mm (range 0-4 mm) and mean angle deviation of osteotomy 3.2° (range 0-10°). The wound healing problem occurred in one patient. No complication was found postoperatively at a mean follow-up of 18.7 months (Table 1). Lung metastasis occurred in one patient at 15 months after surgery and died of the disease due to systematic progression. Two patients were alive with no evidence of the disease. Sequential X-rays at 3-month intervals showed no loosening, no hardware failure of the reconstruction, and no evidence of loosening or fracture.

## 4. Discussion

Primary sacral tumors including osteosarcoma and chordoma are resistant to radiotherapy, which makes surgical resection with negative margins remain to be the treatment choice [1]. R0 resection is vital to achieve good local recurrence [4]. However, resection with negative margin is a technical challenge due to complexity of local anatomy and extensive intraoperative hemorrhage. En bloc sacrectomy was the recommended procedure for the primary sacral tumor involving the upper sacrum [18, 19]. Following en bloc sacrectomy, reconstruction of the spinopelvic continuity is of significance, by which early ambulation and less complications can be realized [7]. Nevertheless, the reconstruction techniques remain controversial [13].

**Figure 5:**
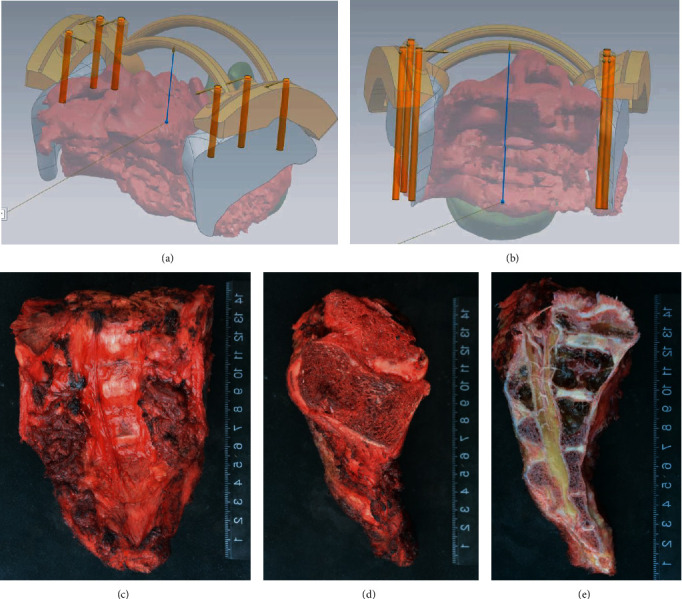
Depth measurement at osteotomy and correlated specimen. Depth of osteotomy was measured preoperatively, and the osteotomy slot was designed accordingly (a, b). The gross specimen (c–e). Lateral view of the specimen showed the osteotomy (d).

The traditional reconstruction method using the screw-rod system was unsatisfactory due to its late complications, especially mechanical failure. Compared with conventional methods using the screw-rod system, 3D printing prosthesis could overcome the problem of low structural strength and reconstruct the lumbosacral and pelvic ring by integrating SPF, PPRF, and ASCF in one step. However, the time-consuming manufacture (usually 4-8 weeks) makes it impracticable because the time interval between neoadjuvant chemotherapy and surgery of primary malignant sacral neoplasms is about 2~3 weeks. In order to decrease the surgical preparation time preoperatively, ready-made 3D-printing prosthesis was used for reconstruction. However, mechanical failures such as prosthetic loosening were also observed in patients who underwent reconstruction with a 3D-printed prosthesis after total sacrectomy due to inadequate bone-implant contact, which was essential for osteointegration. The inadequate bone-implant contact was partially caused by inaccurate osteotomy, which was commonly seen when using the Gigli saw lateral to the sacroiliac joint. A patient-specific surgical jig with osteotomy slots can facilitate precise osteotomy using either the Gigli saw (two-whole passing Gigli saw) or oscillating saw (groove design). The site and direction can be indicated by the jig. With the patient-specific surgical jig, the oscillating saw can be used to perform the osteotomy at planned planes. By this way, the bony cutting surfaces can match the bilateral iliac contact surfaces of the endoprosthesis precisely. This was of great help in promoting bone ingrowth and decreases the risk of mechanical failures due to inadequate osteointegration. And the depth of osteotomy can be determined according to the preoperative CT and MRI (Figure 5). Therefore, we optimized the workflow to use the patient-specific surgical jig and ready-made 3D-printed prosthesis to achieve accurate surgical resection with adequate margin and surface matching of bone-implant contact.

**Figure 6:**
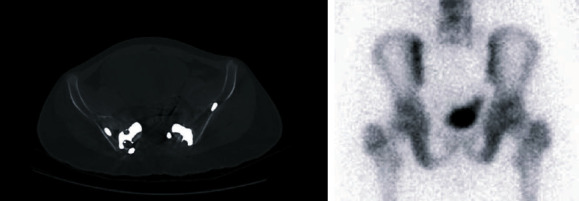
Interface of bone-implant by the CT scan. CT scan during follow-up at 12 months in the patient (patient no. 3) with sacral Ewing's sarcoma. No radiolucence was observed at bone-implant interface. Also, the bone scan showed higher bone metabolism at the interface, which may be caused by the compression provided by the fixation screws.

The advantages of the patient-specific surgical jig and 3D-printed prosthesis included the following: (1) the patient-specific surgical jig was of help in carrying out the osteotomy with planned margins and facilitating prosthetic reconstruction with perfect surface matching of bony cut and implant and (2) the prosthesis consisted of SPF, PPRF, and ASCF, the three essential structures in the lumbosacral region, and efficient reconstruction can be conducted [20]. The prosthesis was connected to the pelvis and lumbar by fixation screws and pedicle screw-rods, making reconstruction convenient and feasible. (3) The porous surface on the 3D-printed prosthesis provided potential osteointegration achieving long-term stability (Figure 6). Computer-assisted surgery (CAS), such as intraoperative navigation and patient-specific instrumentation (PSI), was initially used to improve accuracy of implant placement, osteotomy, and tumor margin. A cadaveric study [21] showed no difference between CAS+PSI and PSI resections in location accuracy, while both strategies were reported to achieve a mean local accuracy of less than 2 mm. Navigation may facilitate surgical jig placement to compensate for the potential limitation of the inaccurate fit to the bone.

Previously, no surgical jig was used intraoperatively [15] and inadequate matching of bone-implant contact at bilateral iliac osteotomy might occur, which was correlated with mechanical failure due to inadequate bone ingrowth. In the present study, we optimized the workflow to design and manufacture a patient-specific surgical jig and ready-made 3D-printed prosthesis for en bloc sacrectomy and reconstruction. By this strategy, accurate osteotomy can be achieved facilitating both achieving negative surgical margin and 3D-printed endoprosthesis reconstruction by improving the surface contact and bone ingrowth at bony cut and metallic porous structures. Comprehensively, the patient-specific surgical jig and ready-made 3D-printed prosthesis are options to be considered for en bloc sacrectomy and reconstruction.

## Figures and Tables

**Table 1:** Demographics of the 3 patients.

No.	Age/sex	Diagnosis	Chemotherapy	Operation time	Estimated blood loss	Follow-up time	Outcome
1	20/M	Osteosarcoma	MAPI	150	3600	19	DOD
2	45/M	Chordoma	No	190	4000	24	NED
3	13/M	Ewing's sarcoma	VAC-IE	190	3600	13	NED

MAPI: methotrexate, doxorubicin, cisplatin, ifosfamide; VAC-IE: doxorubicin, vincristine, cyclophosphamide, ifosfamide, etoposide; DOD: dead of disease; NED: no evidence of disease.

## Data Availability

The data used to support the findings of this study are included within the article.

## Supplementary Materials

Supplementary Materials The intraoperative video showed how the osteotomy was performed using oscillating saw and surgical jig.
